# Metabolic reprogramming in plant defense: linking signaling networks to metabolomics-driven insights

**DOI:** 10.1080/15592324.2026.2672221

**Published:** 2026-05-13

**Authors:** Shahrukh Khan, Zeenat Korai, Liting Yang, Shakal Khan Korai, Shengnan Li, Usman Zulfiqar, Mohammed S. Alotaibi, Dilnozakhon Asadullaeva, Muydinjon Muminov, Mayank Anand Gururani, Xiaoshan Wang

**Affiliations:** a College of Animal Science and Technology, Yangzhou University, Yangzhou, People's Republic of China; b Jiangsu Yancheng Wetland Rare Birds National Nature Reserve, Yancheng, People's Republic of China; c Department of Agronomy, Faculty of Agriculture and Environment, The Islamia University of Bahawalpur, Bahawalpur, Pakistan; d Department of Biology, Nakhchivan State University, Nakhchivan, Azerbaijan; e Department of Clinical Laboratories Sciences, Turabah University College, Taif University, Taif, Saudi Arabia; f Department of Forestry and Landscape Design, Tashkent State Agrarian University, Tashkent, Uzbekistan; g Department of Chemistry, Andijan State University, Andijan, Uzbekistan; h Department of Startup Projects, Kokand University Andijan Branch, Andijan, Uzbekistan; i Department of Biology, College of Science, United Arab Emirates University, Al Ain, United Arab Emirates

**Keywords:** Plant defense signaling, metabolic reprogramming, phytohormone crosstalk, central carbon metabolism, specialized metabolism, growth-defense trade-off, metabolomics, multi-omics integration

## Abstract

Plants adapt to biotic and abiotic stresses through extensive metabolic reprogramming that reallocates cellular resources toward defense while maintaining metabolic homeostasis. This process is tightly regulated by interconnected signaling networks, in which phytohormones, including salicylic acid, jasmonic acid, ethylene, and abscisic acid, integrate stress perception with transcriptional and metabolic responses. Recent advances in metabolomics have enabled systems-level characterization of these dynamic processes, revealing how metabolic pathways are coordinately rewired during stress adaptation. In this review, we synthesize current understanding of the mechanistic links between early signaling events and downstream metabolic outcomes. We highlight how stress perception through pattern recognition receptors, Ca^2+^ influx, reactive oxygen species, and mitogen-activated protein kinase cascades is coupled to transcriptional and post-transcriptional regulation of both primary and specialized metabolisms. Key metabolic adjustments include reconfiguration of central carbon metabolism, maintenance of redox balance, and induction of defense-associated compounds such as phenylpropanoids, flavonoids, terpenoids, and phytoalexins. We further examine the metabolic basis of the growth-defense trade-off, emphasizing the roles of TARGET OF RAPAMYCIN (TOR) and SNF1-RELATED KINASE 1 (SnRK1) in coordinating energy allocation under stress conditions. Emerging approaches, including targeted and untargeted metabolomics, spatial and temporal profiling, stable isotope-assisted fluxomics, and multi-omics integration, are discussed as key tools for dissecting plant defense metabolism. Despite these advances, challenges remain in linking metabolic changes to causal defense functions and in resolving context-dependent responses under complex stress conditions. Collectively, this review provides a mechanistic and systems-level framework that connects signaling networks with metabolic reprogramming, offering new opportunities for improving crop resilience through metabolic engineering and precision breeding.

## Graphical abstract

**Figure:**
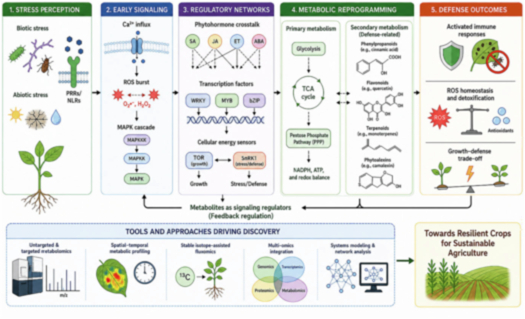

## 1. Introduction

Plants are continuously exposed to a wide range of environmental challenges, including biotic stresses such as pathogens and herbivores, and abiotic stresses such as drought, salinity, temperature extremes, and nutrient limitation. In contrast to mobile organisms, plants cannot escape these adverse conditions and therefore rely on highly dynamic and integrated physiological and biochemical responses to survive. Among these, the capability to rapidly and coordinately reconfigure cellular metabolism has emerged as a central determinant of stress adaptation and resilience.^1-4^

Traditionally, plant stress responses have been studied through the lens of individual pathways or metabolites, often focusing on specific defense compounds or signaling molecules. However, accumulating evidence over the past two decades demonstrates that plant responses to stress involve system-wide metabolic reprogramming, encompassing coordinated adjustments across both primary and secondary metabolic networks. In this review, we define metabolic reprogramming as the regulated and dynamic reallocation of metabolic fluxes, involving (i) remodeling of central carbon and nitrogen metabolism, (ii) induction of specialized metabolite biosynthesis, and (iii) generation of metabolomic signatures associated with stress adaptation. This conceptual shift has been driven in part by advances in high-throughput analytical technologies, enabling the simultaneous detection and quantification of hundreds to thousands of metabolites,^5-9^ and revealing metabolism as a highly interconnected and responsive network rather than a collection of static pathways.

Importantly, metabolic reprogramming is not merely a passive consequence of stress but an actively controlled process that links environmental perception to biochemical outcomes. Early stress signals, including Ca^2+^ influx, reactive oxygen species (ROS) bursts, and mitogen-activated protein kinase (MAPK) cascades, activate downstream regulatory modules such as phytohormones and transcription factors, which in turn control the expression of metabolic genes and enzyme activities. Through this signal-regulator-metabolism axis, plants redirect metabolic fluxes to support defense, for example, by enhancing glycolysis and the tricarboxylic acid (TCA) cycle to meet increased energy demands, while channeling intermediates into the biosynthesis of defense-related compounds such as phenylpropanoids and flavonoids.^10-14^

A defining feature of plant stress adaptation is the integration of metabolic reprogramming with complex regulatory networks involving phytohormones, transcription factors, and signaling cascades. Phytohormones such as salicylic acid (SA), jasmonic acid (JA), ethylene (ET), and abscisic acid (ABA) function as central regulatory hubs that coordinate defense responses and associated metabolic changes. These signaling pathways do not act in isolation; rather, they interact through synergistic and antagonistic crosstalk to fine-tune transcriptional programs and metabolic outputs in a context-dependent manner. For instance, SA- and JA-mediated pathways can differentially regulate branches of secondary metabolism, thereby shaping the composition and function of defense-associated metabolites.^15-17^

Recent advances in metabolomics have provided unprecedented insights into the dynamics of plant metabolic reprogramming under stress. Targeted and untargeted approaches, including liquid chromatography-mass spectrometry (LC-MS), gas chromatography-mass spectrometry (GC-MS), and nuclear magnetic resonance (NMR), enable comprehensive profiling of metabolites across tissues, developmental stages, and environmental conditions.^18-20^ In addition, emerging approaches such as spatial metabolomics and isotope-assisted flux analysis now allow the resolution of temporal and spatial dynamics of metabolic responses, thereby linking metabolic changes more directly to underlying regulatory mechanisms.^21-23^

Despite these advances, a major challenge remains in integrating molecular signaling networks with downstream metabolic outcomes to achieve a systems-level understanding of plant defense. While key signaling components and metabolic pathways have been extensively characterized, their mechanistic connections linking specific signaling pathways and transcriptional regulators to defined metabolic branches and functional defense outputs remain insufficiently resolved. Addressing this gap is essential for understanding how plants coordinate resource allocation between growth and defense, a process governed by metabolic costs, energy availability, and carbon-nitrogen partitioning. ^24-26^

In this context, the present review aims to provide an integrated and mechanistic synthesis of plant defense-associated metabolic reprogramming. We first establish a conceptual framework linking stress perception, signaling networks, and transcriptional regulation to metabolic remodeling. We then examine how major phytohormonal pathways orchestrate specific metabolic outputs, followed by a detailed analysis of reprogramming in primary and secondary metabolism. Particular emphasis is placed on pathway-specific regulation, functional roles of metabolites, and metabolic costs associated with defense activation, including the growth-defense trade-off. Finally, we discuss recent advances in metabolomics and multi-omics integration, including emerging computational and predictive approaches, and highlight key challenges and future directions for translating metabolic insights into crop improvement strategies.

## 2. Conceptual framework of metabolic reprogramming in plant defense

Metabolic reprogramming in plant defense is not a passive downstream consequence of stress perception but a coordinated, multi-layered regulatory process that integrates signaling networks with metabolic responses. Rather than functioning as isolated modules, stress signaling and metabolism operate as an interconnected system in which early molecular events rapidly reshape metabolic fluxes to meet the energetic and biochemical demands of defense. This systems-level perspective is supported by studies showing that metabolism not only responds to stress but also actively contributes to defense regulation.^27-29^ Recent advances in multi-omics and systems biology further highlight that plant immunity involves dynamic redistribution of cellular resources, with metabolism acting both as an effector and regulator of stress adaptation.^30^^,^^31^

**Figure 1.**
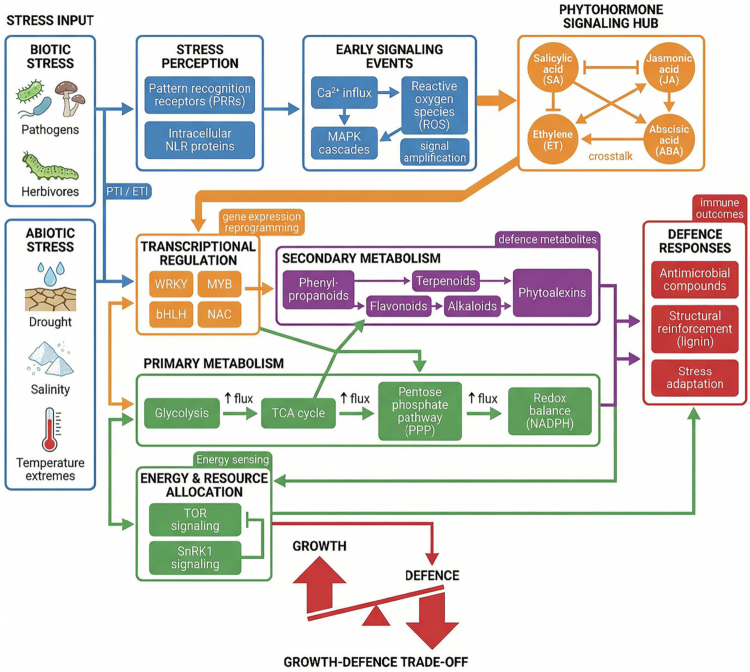
Integrated framework of metabolic reprogramming in plant defense. Environmental stresses (biotic and abiotic) are perceived by pattern recognition receptors (PRRs) and NLR proteins, triggering PTI and ETI and activating early signaling events, including Ca^2+^ influx, ROS production, and MAPK cascades. These signals converge on a phytohormone hub (SA, JA, ET, ABA) that regulates transcription factors (WRKY, MYB, bHLH, NAC), leading to metabolic reprogramming. Primary metabolism is redirected through increased flux in glycolysis, the TCA cycle, and the pentose phosphate pathway to support energy production and redox balance, providing precursors for secondary metabolism. This results in the accumulation of defense metabolites (phenylpropanoids, flavonoids, terpenoids, alkaloids, phytoalexins). Energy sensing via TOR and SnRK1 coordinates resource allocation, shaping the growth-defense trade-off and ultimately determining defense outcomes, including antimicrobial activity, structural reinforcement, and stress adaptation.

A central feature of this framework is the hierarchical yet interconnected cascade linking stress perception to metabolic outcomes: stress perception → signal transduction → regulatory control → metabolic reconfiguration → defense responses (Figure 1). Upon stress detection by pattern recognition receptors (PRRs) or intracellular immune receptors, early signaling events, including Ca^2+^ influx, reactive oxygen species (ROS) bursts, and MAPK cascades, initiate downstream regulatory processes. These signaling pathways converge on phytohormone networks (e.g., SA, JA, ET, ABA) and transcription factors, which collectively regulate genes associated with both primary and secondary metabolisms. This integration ensures that metabolic outputs are precisely aligned with the type, intensity, and temporal dynamics of the stress stimulus.^32^^,^^33^

**Figure 2.**
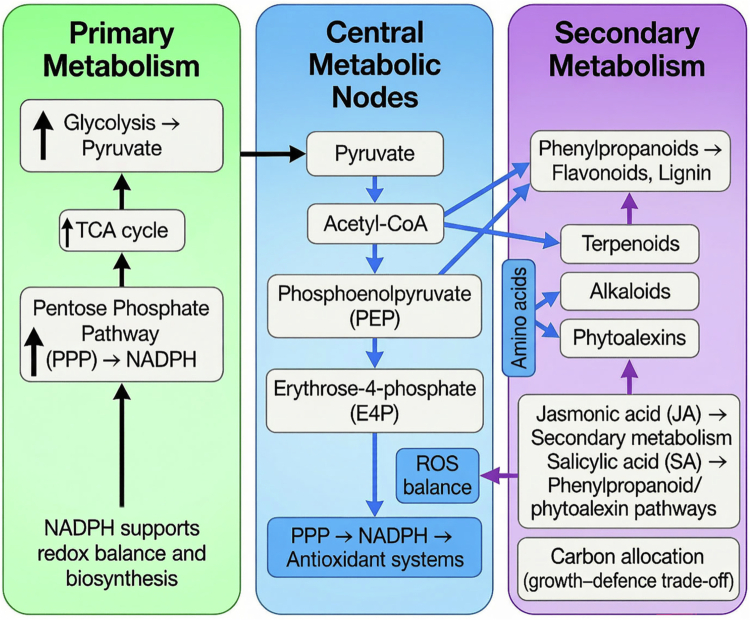
Reprogramming of primary and secondary metabolism during plant defense. Stress-induced metabolic reprogramming enhances flux through glycolysis, the tricarboxylic acid (TCA) cycle, and the pentose phosphate pathway (PPP), generating energy and NADPH for redox balance. Central metabolites, including pyruvate, Acetyl-CoA, phosphoenolpyruvate (PEP), and erythrose-4-phosphate (E4P), link primary metabolism to the biosynthesis of defense-related secondary metabolites. These pathways support the production of phenylpropanoids, terpenoids, alkaloids, and phytoalexins, with additional contributions from amino acid metabolism. Jasmonic acid (JA) and salicylic acid (SA) regulate these processes, while carbon allocation shifts toward defense, reflecting the growth–defense trade-off.

At the core of metabolic reprogramming lies the functional integration of primary and secondary metabolism. Primary metabolic pathways, including glycolysis, the tricarboxylic acid (TCA) cycle, and the pentose phosphate pathway (PPP), provide ATP, reducing equivalents (NADPH), and key intermediates required for defense. These intermediates act as central metabolic nodes linking core metabolism to the biosynthesis of specialized defense compounds such as phenylpropanoids, flavonoids, terpenoids, and alkaloids (Figure 2). Importantly, these metabolic shifts are not uniform but are selectively regulated depending on the signaling context and stress type, reflecting the plasticity of plant metabolic networks.^27^^,^^34^

Another key aspect of this framework is the bidirectional relationship between metabolism and signaling. Metabolites are not only end products of defense responses but can also function as signaling molecules or modulators of cellular redox status, thereby influencing upstream regulatory pathways. For example, changes in ROS levels and NADPH availability act both as metabolic outputs and signaling inputs, reinforcing feedback loops that stabilize or amplify defense responses.^35^^,^^36^

Finally, metabolic reprogramming operates under constraints imposed by resource availability and energetic costs. The diversion of carbon, nitrogen, and energy toward defense-associated pathways often occurs at the expense of growth, forming the basis of the growth–defense trade-off. This balance is regulated by energy-sensing pathways and broader metabolic networks that integrate developmental and stress signals (Figure 1).^24^^,^^37^

Together, this conceptual framework highlights that plant defense should be understood as an integrated system in which signaling networks, regulatory mechanisms, and metabolic pathways operate in a coordinated and dynamic manner. Advances in metabolomics and multi-omics integration have enabled the identification of key regulators and metabolic signatures associated with stress responses (Tables 1 and 2). However, a major challenge remains in establishing direct causal links between specific signaling pathways and defined metabolic outputs, particularly under complex and fluctuating environmental conditions.^35^^,^^38^

**Table 1.** Key molecular regulators of metabolic reprogramming in plant defense.

Category	Component/Gene	Functional role in defense	Metabolic impact	References
Stress perception	PRRs (e.g., FLS2, EFR)	Recognition of PAMPs; initiation of PTI	Triggers downstream signaling leading to metabolic shifts	[40,229]
	NLR proteins (e.g., RPS2, RPM1)	Effector recognition; activation of ETI	Strong metabolic reprogramming; enhanced defense metabolite synthesis	[39,41]
Calcium signaling	Ca^2+^ influx, CDPKs	Early signal transduction	Modulates enzyme activity and metabolic fluxes	[44,230]
ROS signaling	RBOHD, NADPH oxidases	ROS burst and signaling	Redox regulation; activation of antioxidant and PPP pathways	[46,47]
MAPK cascades	MPK3, MPK4, MPK6	Signal amplification; transcriptional regulation	Induction of phenylpropanoid and defense metabolism	[49,231]
Salicylic acid (SA)	ICS1, NPR1	SA biosynthesis and signaling; SAR activation	Activation of phenylpropanoid pathway; redox modulation	[63,82]
Jasmonic acid (JA)	LOX, AOS, AOC, COI1	JA biosynthesis and perception	Induction of terpenoids, alkaloids, and defense metabolites	[87,88]
Ethylene (ET)	EIN2, EIN3, ERFs	Hormonal signaling; defense gene regulation	Synergistic activation of defense metabolism with JA	[92,232]
Abscisic acid (ABA)	PYR/PYL receptors, SnRK2	Abiotic stress signaling; stomatal regulation	Modulates osmolytes, antioxidants, and energy balance	[93,233]
Transcription factors	WRKY (e.g., WRKY33)	Regulation of defense genes	Controls phytoalexin and secondary metabolite biosynthesis	[234]
	MYB, bHLH	Regulation of phenylpropanoid/flavonoid pathways	Controls flavonoid and lignin biosynthesis	[57,58]
	NAC family	Stress-responsive transcriptional control	Modulates lignification and ROS metabolism	[60]
Epigenetic regulation	Histone modifications, DNA methylation	Chromatin remodeling; stress memory	Controls the accessibility of metabolic gene networks	[66,67]
Post-translational regulation	Phosphorylation, ubiquitination	Rapid modulation of protein activity	Adjusts enzyme activity and signaling proteins	[71]
Energy signaling	SnRK1	Energy deficit sensor	Promotes catabolism; supports defense metabolism	[73]
	TOR	Growth regulator under nutrient sufficiency	Promotes anabolic metabolism; suppresses defense	[95,163]
Sugar transport & signaling	SWEET transporters	Sugar efflux and redistribution	Controls carbon allocation during defense	[105,106]
	bZIP, SnRK1-associated TFs	Sugar-responsive transcriptional control	Links energy status with metabolic gene expression	[72]

**Table 2.** Representative metabolomics studies of plant defense under biotic and abiotic stress.

Plant species	Stress type	Metabolomics platform	Key metabolites identified	Major findings	References
*Arabidopsis thaliana*	Pseudomonas syringae (biotic)	LC-MS, GC-MS	Camalexin, SA, amino acids	Induction of phytoalexins and reprogramming of amino acid metabolism during pathogen infection	[134,167]
*Arabidopsis thaliana*	Combined abiotic stress	GC-MS	Sugars, organic acids, and amino acids	Dynamic shifts in central metabolism under stress combinations	[10,235]
Rice (*Oryza sativa*)	Blast fungus (*Magnaporthe oryzae*)	LC-MS	Diterpenoid phytoalexins, flavonoids	Accumulation of phytoalexins linked to resistance responses	[135]
Tomato (*Solanum lycopersicum*)	Drought stress	GC-MS, LC-MS	Sugars, organic acids, phenolics	Reprogramming of carbon metabolism and antioxidant pathways	[236,237]
Wheat (*Triticum aestivum*)	Heat and drought stress	GC-MS	Proline, sugars, TCA intermediates	Osmoprotectant accumulation and metabolic adjustment under stress	[238,239]
Maize (*Zea mays*)	Herbivory	LC-MS	Benzoxazinoids, flavonoids	Induction of defense metabolites and signaling compounds	[240]
Soybean (*Glycine max*)	Fungal infection	LC-MS	Isoflavonoids, phytoalexins	Enhanced secondary metabolism associated with pathogen resistance	[27]
Grapevine (*Vitis vinifera*)	UV/abiotic stress	LC-MS	Resveratrol, flavonoids	Accumulation of phenolic compounds for oxidative protection	[241]
Barley (*Hordeum vulgare*)	Powdery mildew	GC-MS, LC-MS	Phenolics, amino acids	Metabolic shifts associated with defense activation	[242]
Potato (*Solanum tuberosum*)	Pathogen infection	LC-MS	Alkaloids, phenolics	Activation of defense-related secondary metabolites	[18,243]
*Arabidopsis thaliana*	Light stress	LC-MS	Flavonoids, antioxidants	Enhanced ROS scavenging via flavonoid accumulation	[137]
Rice (*Oryza sativa*)	Salinity stress	GC-MS	Sugars, amino acids	Metabolic adjustment supporting osmotic balance and stress tolerance	[244,245]

### 2.1. Stress perception and early signaling events

The initiation of metabolic reprogramming is triggered by the perception of external stress signals through highly conserved surveillance systems. In biotic interactions, pattern recognition receptors (PRRs) detect pathogen-associated molecular patterns (PAMPs), activating pattern-triggered immunity (PTI). In parallel, intracellular nucleotide-binding leucine-rich repeat (NLR) proteins recognize pathogen-derived effectors, leading to effector-triggered immunity (ETI), which is typically stronger and associated with greater metabolic cost.^39-43^ These perception systems establish the first layer of specificity by determining the nature and intensity of downstream metabolic responses.

One of the earliest and most universal responses following stress perception is a rapid increase in cytosolic Ca^2+^ levels. Calcium signatures are decoded by sensor proteins such as calmodulins and calcium-dependent protein kinases (CDPKs), which act as key intermediates linking stress perception to downstream transcriptional and metabolic regulation.^44^^,^^45^ Importantly, Ca^2+^ signaling is not limited to transcriptional control but can directly influence metabolic enzyme activities and mitochondrial function, thereby providing an immediate coupling between signaling events and metabolic adjustment.

Concurrent with Ca^2+^ influx, plants generate a rapid burst of reactive oxygen species (ROS), primarily mediated by NADPH oxidases such as RESPIRATORY BURST OXIDASE HOMOLOG D (RBOHD). While ROS function as antimicrobial agents, they also serve as central signaling molecules that modulate redox-sensitive metabolic pathways.^46-48^ For example, ROS-dependent changes in cellular redox state can regulate flux through the pentose phosphate pathway and antioxidant systems, thereby linking early signaling events to the production of reducing equivalents (NADPH) required for defense-associated metabolism. This highlights the dual role of ROS as both signaling intermediates and regulators of metabolic reconfiguration.

These early signals are further integrated and amplified by mitogen-activated protein kinase (MAPK) cascades. MAPKs such as MPK3, MPK4, and MPK6 transmit extracellular signals to the nucleus, where they regulate transcription factors and, in some cases, metabolic enzymes.^49^^,^^50^ Through this regulatory layer, MAPK signaling provides a mechanistic bridge between early stress perception and the transcriptional activation of metabolic pathways. Notably, MAPK cascades have been implicated in the induction of phenylpropanoid biosynthesis and other defense-related metabolic processes, illustrating how signaling pathways can selectively direct metabolic outputs.

Beyond these canonical signaling pathways, recent studies indicate that early stress responses are accompanied by rapid changes in cellular energy status. Alterations in ATP levels and redox balance can occur within minutes of stress perception, suggesting that metabolic adjustments are initiated in parallel with, rather than subsequent to, signaling events.^51^^,^^52^ This observation challenges the classical linear model of “signaling first, metabolism later” and instead supports a model of tight and reciprocal coupling between signaling and metabolic processes. Collectively, Ca^2+^ signaling, ROS dynamics, and MAPK cascades form an integrated network that rapidly translates stress perception into metabolic reprogramming. Through coordinated regulation of transcriptional activity, enzyme function, and redox balance, these early signaling events ensure that metabolic responses are both rapid and context-specific, enabling plants to tailor defense strategies to distinct biotic and abiotic challenges.^53^^,^^54^

### 2.2. Transcriptional and post-transcriptional regulation

The transition from early signaling events to sustained metabolic reprogramming is mediated by tightly controlled transcriptional and post-transcriptional regulatory mechanisms. At this stage, stress-induced signals are translated into specific metabolic outputs through the activation of transcription factors (TFs) that directly regulate genes associated with primary and secondary metabolism.

Among these, WRKY transcription factors are central regulators of plant immunity and metabolic reprogramming. For example, WRKY33 directly controls genes in tryptophan-derived pathways, thereby promoting the biosynthesis of defense metabolites such as camalexin.^55^^,^^56^ Similarly, MYB and bHLH transcription factors regulate phenylpropanoid and flavonoid biosynthesis, linking stress signaling to the production of antimicrobial and antioxidant compounds.^57-59^ NAC transcription factors further contribute by modulating pathways such as lignin biosynthesis and reactive oxygen metabolism, reinforcing structural and redox-based defense mechanisms.^60^^,^^61^ These examples highlight how distinct TF families target specific metabolic branches, ensuring pathway-specific regulation rather than a uniform metabolic response.

A key regulatory hub integrating signaling and transcriptional control is NONEXPRESSOR OF PATHOGENESIS-RELATED GENES 1 (NPR1). NPR1 mediates salicylic acid (SA)-dependent transcriptional responses through interaction with TGA transcription factors and regulates a broad set of defense-related genes, including those involved in phenylpropanoid metabolism.^62-65^ This regulatory module exemplifies how hormone signaling pathways are directly coupled to transcriptional activation of metabolic processes.

Beyond transcriptional control, epigenetic mechanisms provide an additional layer of regulation. Histone modifications and DNA methylation influence chromatin accessibility, thereby modulating the expression of defense- and metabolism-related genes.^66-68^ Such mechanisms enable dynamic tuning of metabolic responses and contribute to stress memory, allowing plants to respond more efficiently to recurring stress conditions (Table 1).

Post-transcriptional regulation further refines metabolic reprogramming through control of RNA processing, stability, and translation. Small RNAs and RNA-binding proteins regulate the expression of key metabolic enzymes and signaling components under stress conditions.^69^^,^^70^ In parallel, post-translational modifications including phosphorylation, ubiquitination, and redox-based changes enable rapid adjustment of enzyme activities and signaling proteins, providing a fast and reversible mechanism for metabolic control.^54^^,^^71^

Importantly, increasing evidence indicates that transcriptional and metabolic networks operate within feedback-regulated circuits. Metabolites themselves can influence gene expression, acting as signaling molecules that regulate transcription factors such as bZIPs and energy-sensing regulators associated with SnRK1.^72-74.^ This reciprocal interaction ensures that metabolic state is not only an output but also a regulator of transcriptional programs, enabling fine-tuned and context-dependent metabolic responses. Overall, transcriptional and post-transcriptional regulation represent the central interface through which early signaling events are translated into coordinated metabolic reprogramming. Rather than functioning as linear pathways, these regulatory layers form dynamic and interconnected networks that allow precise modulation of metabolic outputs while maintaining cellular homeostasis under stress conditions.

## 3. Hormonal control of defense-associated metabolic reprogramming

Phytohormones serve as central integrators of plant defense, linking stress perception and signaling networks to transcriptional regulation and downstream metabolic reprogramming.^75^ Rather than acting as isolated signaling molecules, hormones operate within highly interconnected networks that modulate metabolic fluxes and enable plants to dynamically balance defense activation with growth and energy homeostasis.^76^ Importantly, hormone-mediated signaling extends beyond transcriptional control and directly influences both primary and secondary metabolic pathways, thereby shaping the biochemical landscape of stress responses.^77-80^

A defining feature of hormone-driven metabolic reprogramming is its context dependency. The nature of the stress determines not only which hormonal pathways are activated but also how these pathways interact to regulate specific metabolic branches. Salicylic acid (SA) is primarily associated with defense against biotrophic pathogens, whereas jasmonic acid (JA) and ethylene (ET) mediate responses to necrotrophic pathogens and herbivory.^81^ In contrast, abscisic acid (ABA), although classically linked to abiotic stress, plays a crucial role in modulating defense-associated metabolic changes, particularly under combined or sequential stress conditions.

These hormonal pathways converge on metabolic networks through the regulation of transcription factors, enzyme activities, and resource allocation processes. For example, hormone signaling can redirect carbon flux from growth-related pathways toward the biosynthesis of defense metabolites, while simultaneously modulating redox balance and energy status. Such coordinated regulation ensures that metabolic outputs are tailored to specific stress conditions, rather than representing a uniform defense response. ^53^

Moreover, interactions among hormonal pathways often involve synergistic or antagonistic crosstalk add layer of regulation to metabolic reprogramming. These interactions influence not only defense gene expression but also the composition and accumulation of specific metabolite classes, thereby determining the effectiveness and specificity of plant defense responses.

### 3.1. Salicylic acid (SA) pathway

Salicylic acid (SA) signaling represents a central regulatory module linking immune activation to metabolic reprogramming, particularly in responses to biotrophic pathogens that require sustained and systemic defense. SA is primarily synthesized via the isochorismate pathway, catalyzed by ISOCHORISMATE SYNTHASE 1 (ICS1), which connects its biosynthesis to primary metabolism through the shikimate pathway.^82^^,^^83^ This metabolic origin highlights a direct interface between central carbon metabolism and hormone production, positioning SA as both a signaling molecule and a metabolic output.

A key mediator of SA signaling is the redox-sensitive regulator NONEXPRESSOR OF PATHOGENESIS-RELATED GENES 1 (NPR1), which controls transcriptional reprogramming of defense-associated genes. Upon SA accumulation, NPR1 undergoes redox-dependent monomerization and translocates to the nucleus, where it interacts with TGA transcription factors to activate systemic acquired resistance (SAR) and downstream metabolic pathways.^62^^,^^63^ Through this SA–NPR1–TGA regulatory module, signaling is directly coupled to the activation of metabolic genes, particularly those involved in phenylpropanoid biosynthesis. This leads to the accumulation of defense-related metabolites such as lignin, flavonoids, and other antimicrobial compounds.^84^

Beyond secondary metabolism, SA signaling also drives significant changes in primary metabolic pathways. Metabolomic analyses have shown that SA enhances flux through the pentose phosphate pathway, thereby increasing NADPH production required for redox homeostasis and biosynthetic processes associated with defense.^51^^,^^85^ In addition, SA signaling is associated with shifts in amino acid and organic acid metabolism, reflecting a broader reallocation of resources toward anabolic pathways that support the synthesis of defense metabolites. These coordinated adjustments illustrate how SA signaling reprograms both central and specialized metabolism to sustain defense responses.

SA also plays a critical role in systemic signaling, coordinating metabolic reprogramming beyond the site of infection. Mobile signaling molecules such as methyl salicylate and azelaic acid contribute to the establishment of systemic acquired resistance (SAR), enabling distal tissues to undergo defense priming and metabolic adjustment.^86^ This systemic dimension highlights the role of SA in integrating local signaling events with whole-plant metabolic coordination, ensuring a sustained and efficient defense strategy.

### 3.2. Jasmonic acid (JA) pathway

Jasmonic acid (JA) is a central regulator of defense against necrotrophic pathogens and herbivorous insects and plays a dominant role in coordinating defense-associated metabolic reprogramming. JA is synthesized via the octadecanoid pathway, involving enzymes such as lipoxygenase (LOX), allene oxide synthase (AOS), and allene oxide cyclase (AOC).^87^ This biosynthetic route originates from membrane-derived fatty acids, thereby linking lipid metabolism directly to stress signaling and defense activation.

Perception of JA is mediated by the CORONATINE INSENSITIVE 1 (COI1) receptor, a component of the SCF^COI1 ubiquitin ligase complex. Binding of the bioactive form jasmonoyl-isoleucine (JA-Ile) promotes the degradation of JAZ repressors, resulting in the release and activation of transcription factors such as MYC2.^88^ Through the COI1–JAZ–MYC2 regulatory module, JA signaling is directly coupled to transcriptional activation of genes involved in specialized metabolism. This includes pathways responsible for the biosynthesis of terpenoids, alkaloids, and glucosinolates, which function as key defense compounds.

JA-mediated metabolic reprogramming is particularly evident in the induction of secondary metabolite pathways that provide both direct and indirect defense. For instance, JA signaling enhances the production of volatile terpenes that attract natural enemies of herbivores, representing an indirect defense strategy.^89^ In addition, JA regulates the accumulation of glucosinolates and alkaloids, which act as toxic or deterrent compounds against herbivores and pathogens.^90^ These pathway-specific outputs highlight the role of JA in tailoring metabolic responses to biotic stress conditions distinct from SA-mediated defense.

Beyond secondary metabolism, JA signaling also drives substantial changes in primary metabolic processes. Activation of JA responses is associated with reallocation of carbon and nitrogen resources from growth-related pathways toward defense-associated metabolism. This includes modulation of sugar metabolism and amino acid biosynthesis, reflecting the metabolic cost of sustained defense activation.^91^ Increased respiratory activity and energy demand are frequently observed during JA responses, underscoring the importance of metabolic reprogramming in maintaining defense functions. Moreover, JA signaling exemplifies how hormone-mediated pathways can selectively reprogram both primary and secondary metabolism to support defense against necrotrophic stress, while simultaneously imposing high energetic and resource costs on the plant.

### 3.3. Ethylene, ABA, and hormonal crosstalk

Ethylene (ET) and abscisic acid (ABA) play critical roles in modulating defense-associated metabolic reprogramming, largely through their interactions with salicylic acid (SA) and jasmonic acid (JA) signaling pathways. Ethylene frequently acts synergistically with JA during responses to necrotrophic pathogens, enhancing the expression of defense-related genes and promoting the accumulation of antimicrobial metabolites.^92^ This synergistic interaction is mediated by transcriptional regulators such as ETHYLENE INSENSITIVE 3 (EIN3) and ETHYLENE RESPONSE FACTORs (ERFs), which integrate hormonal signals with the activation of genes involved in secondary metabolism.

In contrast, ABA has a more complex and context-dependent role. While it is primarily associated with abiotic stress responses, ABA also significantly influences defense-related metabolic processes. ABA signaling regulates stomatal closure, osmolyte accumulation, and antioxidant metabolism, thereby contributing to stress tolerance through metabolic adjustments.^93^ However, ABA can also antagonize SA-dependent defense pathways, leading to a trade-off between abiotic stress tolerance and resistance to biotrophic pathogens.^94^ This antagonism reflects a shift in metabolic prioritization, where resources are redirected toward stress adaptation rather than pathogen defense.

Hormonal crosstalk represents a key mechanism by which plants fine-tune metabolic reprogramming under complex and fluctuating environmental conditions. Interactions between SA and JA pathways are often antagonistic, enabling prioritization of defense strategies against specific pathogen types, whereas JA–ET interactions typically act synergistically to enhance secondary metabolite production.^15^ These interactions are mediated through shared transcription factors, signaling components, and metabolic intermediates, allowing coordinated regulation of both defense gene expression and metabolic outputs.

Beyond secondary metabolism, hormonal crosstalk also extends to the regulation of primary metabolism and energy signaling. Central regulators such as TARGET OF RAPAMYCIN (TOR) and SNF1-RELATED KINASE 1 (SnRK1) integrate hormonal cues with cellular energy status to balance growth and defense. TOR promotes anabolic metabolism and growth under favorable conditions, whereas SnRK1 is activated under stress to induce catabolic pathways and energy conservation.^73^^,^^95^ Through these regulatory hubs, hormonal signaling directly influences metabolic homeostasis and resource allocation during defense responses.

Recent metabolomics studies further demonstrate that hormonal interactions generate distinct metabolic profiles that cannot be predicted from individual hormone responses alone.^53^^,^^96^ This emergent behavior highlights the importance of integrative regulatory networks, in which hormone signaling and metabolism are tightly interconnected to produce context-specific defense outcomes. Furthermore, ethylene, ABA, and their interactions with SA and JA form a dynamic regulatory network that coordinates signaling, transcriptional activity, and metabolic reprogramming, enabling plants to optimize defense responses while maintaining metabolic balance under diverse stress conditions.

## 4. Reprogramming of primary metabolism during defense

Defense activation imposes substantial energetic and biosynthetic demands on plant cells, necessitating rapid and coordinated reprogramming of primary metabolism. Rather than serving as a passive supply system, central metabolic pathways actively support immune responses by providing energy (ATP), reducing equivalents (NADPH), and metabolic intermediates required for defense-associated biosynthesis. ^97^

Primary metabolism is dynamically rewired under stress conditions, involving coordinated changes in carbon allocation, redox balance, and energy signaling.^27^^,^^28^^,^^51^^,^^98^^,^^99^ These adjustments enable plants to redirect metabolic fluxes from growth-related processes toward defense, reflecting a fundamental shift in resource prioritization. Importantly, this reprogramming involves not only quantitative changes in metabolic flux but also qualitative reorganization of pathway utilization, allowing plants to rapidly adapt to different stress conditions.

A central feature of this metabolic shift is the prioritization of defense over growth, where carbon and energy resources are reallocated to support the synthesis of defense compounds and maintenance of cellular homeostasis. This reallocation is tightly regulated by hormonal signaling networks and transcriptional control mechanisms, which coordinate metabolic activity with defense requirements. Through this integration, primary metabolism provides the biochemical and energetic foundation necessary for downstream activation of specialized metabolic pathways (Figure 2).

### 4.1. Carbon reallocation and central metabolic fluxes

One of the earliest metabolic responses to stress is the reallocation of carbon resources from growth-associated processes toward defense. This shift is reflected in coordinated changes in central metabolic pathways, particularly glycolysis and the tricarboxylic acid (TCA) cycle, which are reprogrammed to meet both energetic and biosynthetic demands.

Under stress conditions, glycolytic flux is often enhanced, supporting increased respiratory activity and generating key intermediates required for downstream biosynthesis.^38^^,^^100^ Metabolites such as phosphoenolpyruvate (PEP) and pyruvate serve as critical nodes linking glycolysis to the synthesis of amino acids and secondary metabolites, thereby directly connecting central metabolism with defense-related pathways.

The TCA cycle exhibits significant metabolic flexibility during defense responses. In addition to its role in ATP production, the TCA cycle provides carbon skeletons for the biosynthesis of amino acids and specialized metabolites. Accumulation of organic acids such as citrate and malate has been observed under both pathogen infection and abiotic stress, indicating a reorganization of mitochondrial metabolism to support defense-associated biosynthetic processes.^101^^,^^102^ These intermediates function not only as metabolic substrates but also as signals influencing cellular redox balance and metabolic flux distribution.

A key component of carbon reallocation is the activation of the pentose phosphate pathway (PPP), which generates NADPH required for reductive biosynthesis and antioxidant defense. Enhanced PPP activity supports both ROS detoxification and the biosynthesis of defense compounds, highlighting its central role in linking carbon metabolism to redox homeostasis.^103^^,^^104^ This shift underscores the importance of redox metabolism as a driving force in defense-associated metabolic reprogramming.

In addition to intracellular metabolic adjustments, carbon reallocation also occurs at the whole-plant level. Stress conditions alter source-sink relationships, leading to increased mobilization of sugars toward infected or stressed tissues. Sugar transporters, including members of the SWEET family, mediate this redistribution and play key roles in regulating carbon flux during both biotic and abiotic stress.^105^^,^^106^ Such systemic reallocation ensures that metabolic resources are preferentially directed toward defense sites, enabling coordinated responses across tissues.

### 4.2. Energy metabolism and redox homeostasis

Effective defense responses require not only carbon skeletons but also substantial energy input and precise regulation of cellular redox status. Stress conditions are typically associated with increased respiratory activity, reflecting elevated ATP demand to support biosynthesis, cellular maintenance, and defense-related processes. Mitochondria play a central role in this adjustment by modulating respiratory flux and electron transport chain activity to meet changing energy requirements.^107^

At the same time, chloroplast metabolism undergoes significant reprogramming, particularly under conditions where photosynthesis is impaired. Although photosynthetic carbon assimilation often declines during stress, chloroplasts remain essential for defense by generating signaling molecules, redox equivalents, and metabolic intermediates. This dual role highlights the tight coordination between energy metabolism and defense signaling across organelles.^108^

Redox balance represents a critical component of metabolic reprogramming during defense. Stress-induced production of reactive oxygen species (ROS) requires efficient antioxidant systems, including the glutathione and ascorbate pathways, which depend on NADPH generated primarily through the pentose phosphate pathway and associated metabolic routes.^104^ The balance between ROS production and detoxification establishes a dynamic redox environment that influences both signaling pathways and metabolic fluxes.

Importantly, redox status directly regulates the activity of key metabolic enzymes and transcription factors, thereby linking metabolic state to gene expression. Redox-sensitive modifications, particularly thiol-based regulation, enable rapid and reversible control of enzyme activity in central metabolic pathways, allowing flexible adjustment of metabolic flux under stress conditions.^109^ Together, energy metabolism and redox homeostasis form an integrated regulatory system that supports defense responses by coordinating ATP production, metabolic flux, and signaling processes. This integration ensures that defense activation is sustained while maintaining cellular stability under fluctuating environmental conditions.

### 4.3. Sugar signaling, transport, and energy-sensing pathways

Sugars function not only as metabolic substrates but also as key signaling molecules that coordinate growth, metabolism, and defense responses. Changes in sugar availability under stress are sensed by signaling pathways that regulate gene expression and metabolic activity, thereby linking cellular energy status to defense-associated metabolic reprogramming.

Central to this regulatory network are energy-sensing kinases such as SNF1-RELATED KINASE 1 (SnRK1) and TARGET OF RAPAMYCIN (TOR), which integrate metabolic and environmental signals.^73^^,^^95^ SnRK1 is activated under energy-limiting conditions and promotes catabolic processes while repressing energy-intensive biosynthetic pathways. Through this mechanism, SnRK1 stimulates carbon mobilization and supports defense-related metabolism during stress. In contrast, TOR promotes anabolic metabolism and growth under favorable conditions but is typically suppressed during stress, reflecting a shift toward defense prioritization.^110^ The antagonistic relationship between SnRK1 and TOR provides a central regulatory axis that balances growth and defense through metabolic control.

Sugar transporters, including members of the SWEET family and sucrose transporters, play essential roles in regulating carbon distribution and signaling during defense. These transport systems contribute to the redistribution of sugars toward infected or stressed tissues, supporting local metabolic demands for defense. However, they also represent points of vulnerability, as pathogens can exploit sugar transporters to access host resources.^105^^,^^111^ This dual role highlights the importance of tightly regulated sugar transport in determining the outcome of plant-pathogen interactions.

Beyond transport, sugar signaling directly influences transcriptional networks that coordinate primary and secondary metabolism. Sucrose and glucose signaling pathways interact with hormonal networks to regulate defense gene expression and metabolic fluxes, thereby integrating energy status with stress responses.^72^ Through this coordination, sugar signaling ensures that metabolic reprogramming is aligned with both cellular energy availability and environmental conditions.

## 5. Secondary metabolism and defense-associated metabolites

While primary metabolism provides the energetic and biosynthetic foundation for defense, it is the reprogramming of secondary (specialized) metabolism that determines the biochemical specificity and functional outcomes of plant stress responses. Secondary metabolites encompass a diverse array of compounds, including phenylpropanoids, flavonoids, terpenoids, alkaloids, and phytoalexins, which contribute to antimicrobial defense, signaling, structural reinforcement, and ecological interactions. ^112^^,^^113^

Importantly, secondary metabolism should not be considered a uniform downstream output of stress responses, but rather a collection of pathway-specific processes that are selectively activated depending on the nature of the stress and regulatory context. These pathways are tightly controlled by phytohormone signaling networks and transcription factors, enabling plants to generate tailored metabolic responses under biotic and abiotic stress conditions. ^112^^,^^113^

A defining feature of defense-associated secondary metabolism is its close integration with primary metabolic pathways. Central metabolism supplies carbon skeletons, ATP, and reducing equivalents (NADPH), which are redirected into specialized biosynthetic pathways during stress. For example, intermediates derived from glycolysis and the shikimate pathway serve as precursors for phenylpropanoid and flavonoid biosynthesis, while Acetyl-CoA and isoprenoid intermediates support terpenoid production.^114^^,^^115^ This metabolic coupling enables rapid and coordinated synthesis of defense compounds while maintaining cellular homeostasis.

Such integration allows plants to dynamically tailor their metabolic outputs to specific environmental challenges, resulting in distinct metabolite profiles associated with different stress conditions. Metabolomics studies have consistently shown that biotic and abiotic stresses induce highly specific and temporally dynamic metabolite signatures, reflecting the plasticity of plant metabolic networks.^116-120^ This diversity and adaptability of secondary metabolism underpin the effectiveness of plant defense strategies, linking upstream signaling and metabolic reprogramming to functional defense outcomes.

### 5.1. Phenylpropanoids and flavonoids

The phenylpropanoid pathway represents one of the most extensively characterized and metabolically significant defense-associated pathways in plants. It is initiated by the deamination of phenylalanine via PHENYLALANINE AMMONIA-LYASE (PAL), leading to the production of a diverse array of metabolites, including lignin, flavonoids, and hydroxycinnamic acid derivatives.^121^^,^^122^ These compounds contribute to plant defense through multiple mechanisms, including direct antimicrobial activity, reinforcement of cell walls, and modulation of oxidative stress.

Importantly, the phenylpropanoid pathway is tightly regulated at the transcriptional level by MYB, bHLH, and WD40 transcription factor complexes, which control the expression of key biosynthetic genes and enable pathway-specific responses to different stress conditions.^58^^,^^113^^,^^123^ This regulatory framework ensures that distinct branches of the pathway are selectively activated depending on the nature of the stress stimulus.^59^^,^^124^^,^^125^

Flavonoids, synthesized through enzymes such as CHALCONE SYNTHASE (CHS), play central roles in plant defense under both biotic and abiotic stress conditions. Beyond their well-established antioxidant function in scavenging reactive oxygen species (ROS), flavonoids also act as signaling molecules that modulate gene expression, hormone signaling pathways, and stress-responsive networks. Recent studies highlight their involvement in regulating cellular redox balance, mediating stress signaling, and contributing to adaptation under drought, high light, and pathogen attack.^126^^,^^127^ This multifunctional role positions flavonoids as key integrators of metabolic and signaling networks in plant defense.

Lignin biosynthesis represents another critical branch of the phenylpropanoid pathway, contributing to structural defense by reinforcing cell walls and limiting pathogen penetration. Stress-induced lignification is a common response to both biotic and abiotic challenges and reflects a substantial metabolic investment, requiring coordinated reallocation of carbon and reducing power from primary metabolism.^128^ This highlights the strong coupling between phenylpropanoid metabolism and central metabolic pathways, particularly in terms of carbon flux and redox balance.

Metabolomics approaches have provided significant insights into the dynamic regulation of phenylpropanoid metabolism. Untargeted LC-MS profiling has revealed rapid and stress-specific accumulation of flavonoids and related metabolites following pathogen infection in Arabidopsis and crop species, often correlating with enhanced resistance phenotypes.^129^^,^^130^ These findings demonstrate that phenylpropanoid metabolism is highly responsive and finely regulated, producing distinct metabolite signatures that reflect both the type and intensity of stress.

### 5.2. Alkaloids, terpenoids, and phytoalexins

Beyond phenylpropanoids, plants produce a wide range of specialized metabolites that contribute to defense, including terpenoids, alkaloids, and phytoalexins. These metabolite classes are synthesized through distinct but interconnected biosynthetic pathways and play critical roles as antimicrobial agents, deterrents, and signaling molecules in plant defense.

Terpenoids represent the largest class of plant secondary metabolites and are derived from isoprenoid precursors through the mevalonate (MVA) and methylerythritol phosphate (MEP) pathways. These pathways are tightly regulated and closely linked to primary metabolism through the supply of precursors such as Acetyl-CoA and glyceraldehyde-3-phosphate. Terpenoids function in both direct and indirect defense, including toxicity against pathogens and herbivores, as well as the emission of volatile compounds that attract natural enemies of herbivores.^131^ Importantly, jasmonic acid (JA) signaling plays a central role in regulating terpenoid biosynthesis through transcriptional activation of pathway-specific genes, linking stress perception to metabolite production.

Alkaloids constitute a diverse group of nitrogen-containing compounds with well-established defensive functions. Their biosynthesis involves complex pathways integrating amino acid metabolism with specialized enzymatic reactions, highlighting the close interplay between primary and secondary metabolism.^132^ Examples such as nicotine and morphine illustrate how alkaloid production is tightly regulated and often induced under stress conditions, providing effective chemical defense against herbivores and pathogens. The diversity of alkaloid structures reflects their wide range of ecological and physiological roles in plant defense.

Phytoalexins are inducible antimicrobial compounds that are synthesized de novo in response to pathogen attack and represent a hallmark of dynamic metabolic reprogramming. Unlike constitutive defense metabolites, phytoalexin production is rapidly activated through coordinated signaling and transcriptional regulation following stress perception. Camalexin, an indole-derived phytoalexin in Arabidopsis, is a well-characterized example whose biosynthesis is tightly controlled by transcription factors and defense signaling pathways.^133^ Similarly, crop species such as rice and soybean produce distinct phytoalexins that contribute to pathogen resistance, reflecting species-specific metabolic adaptations.

Recent metabolomics studies have revealed that the accumulation of phytoalexins is often accompanied by broader, system-wide metabolic changes. These include coordinated shifts in amino acid, lipid, and carbohydrate metabolism, indicating that defense-associated secondary metabolism operates within a highly integrated metabolic network rather than as isolated pathways.^134^^,^^135^ Such findings emphasize that specialized metabolite production is embedded within global metabolic reprogramming, linking local defense responses to systemic metabolic adjustments.

### 5.3. Specialized metabolites under abiotic stress: insights from metabolomics

While secondary metabolism is traditionally associated with biotic defense, it also plays a critical role in plant responses to abiotic stress. Environmental stresses such as drought, salinity, and temperature extremes induce the accumulation of specialized metabolites that contribute to osmoprotection, antioxidant defense, and cellular stabilization.^136^ These responses are essential for maintaining cellular integrity and metabolic homeostasis under adverse conditions.

Flavonoids and other phenolic compounds are among the most prominent metabolites induced under abiotic stress. Their accumulation under drought and high-light conditions enhances reactive oxygen species (ROS) scavenging capacity and protects photosynthetic machinery from oxidative damage.^137^^,^^138^ Similarly, terpenoids and carotenoids contribute to membrane stability and photoprotection, reducing oxidative stress and preserving cellular function.^139^^,^^140^ These metabolite classes illustrate how secondary metabolism supports abiotic stress tolerance through both protective and regulatory mechanisms.

Metabolomics has been instrumental in revealing the complexity and dynamics of these responses. Unbiased metabolomic profiling has demonstrated that distinct abiotic stresses generate highly specific metabolic signatures, reflecting differential activation of biosynthetic pathways. Time-resolved analyses further show that metabolic responses are dynamic, with early stress-induced changes often differing substantially from later adaptive phases.^10^^,^^141^^,^^142^ Such temporal resolution highlights the importance of distinguishing immediate stress responses from long-term metabolic acclimation.

In addition to well-characterized metabolites, metabolomics approaches have enabled the identification of previously uncharacterized or low-abundance compounds associated with stress tolerance. Integration of metabolomics with transcriptomics and proteomics has facilitated the identification of candidate genes and biosynthetic pathways, providing new opportunities for functional characterization and metabolic engineering.^143-145^ Collectively, these findings underscore the value of metabolomics as a systems-level approach for linking metabolic phenotypes with underlying molecular mechanisms. By capturing the diversity, specificity, and temporal dynamics of metabolite changes, metabolomics provides critical insights into how plants reorganize biochemical networks to adapt to abiotic stress conditions. ^146^

## 6. Growth-defense trade-off: metabolic cost and homeostasis

Activation of plant defense responses imposes substantial metabolic costs, requiring large-scale reallocation of resources that would otherwise support growth and reproduction. This constraint underlies the growth-defense trade-off, a central concept in plant biology that reflects the need to balance resource allocation between competing physiological processes. Rather than representing a simple antagonistic relationship, this trade-off is increasingly understood as a dynamic and tightly regulated process, modulated by metabolic status, environmental conditions, and signaling networks.^24^^,^^147^

At its core, the growth-defense trade-off is governed by the availability and partitioning of carbon, nitrogen, and energy resources. Defense activation redirects carbon flux from growth-associated processes such as cell wall expansion, photosynthetic investment, and biomass accumulation toward the synthesis of defense-related metabolites. This shift is accompanied by increased demand for ATP and reducing equivalents, reflecting the energetic and redox costs associated with sustained defense responses.^25^^,^^148^ Nitrogen allocation further contributes to this trade-off, as amino acids are diverted from protein synthesis and growth-related functions toward the production of nitrogen-containing defense compounds, including alkaloids and phytoalexins.^149^^,^^150^ Together, these adjustments illustrate how both carbon and nitrogen metabolism are reprogrammed to support defense at the expense of growth.

Importantly, this trade-off is actively regulated by signaling networks, including phytohormones and energy-sensing pathways such as SnRK1 and TOR, which integrate environmental cues with cellular metabolic status.^151-153^ Under stress conditions, activation of defense pathways is often associated with suppression of growth-promoting processes, whereas recovery phases involve re-establishment of metabolic balance and growth. Thus, the growth-defense trade-off represents a coordinated reallocation of metabolic resources rather than a fixed limitation, enabling plants to optimize survival under stress while maintaining long-term fitness.^24^^,^^154^

### 6.1. Carbon-nitrogen balance and resource allocation

The allocation of carbon and nitrogen resources plays a central role in determining the outcome of the growth-defense trade-off. Different classes of defense metabolites impose distinct resource demands: phenylpropanoids and related compounds are predominantly carbon-rich, whereas alkaloids and many phytoalexins require substantial nitrogen investment. The relative availability of these resources, therefore, influences both the type and quantity of defense metabolites produced under specific environmental conditions.^155-157^

Under stress conditions, plants often shift toward a defense-oriented metabolic state by reallocating carbon from growth-associated processes to defense pathways.^27^^,^^37^ This involves redistribution of photosynthetically derived carbon from assimilation and storage pools toward the biosynthesis of defense compounds, accompanied by adjustments in source-sink relationships and mobilization of stored carbohydrates.^72^^,^^98^ In parallel, nitrogen metabolism is reprogrammed to support defense. Nitrogen assimilation and redistribution are redirected toward the synthesis of nitrogen-containing defense compounds and stress-responsive proteins, reflecting coordinated regulation of both carbon and nitrogen metabolic networks.^158^^,^^159^

Importantly, carbon-nitrogen balance is not static but dynamically regulated in response to environmental and nutritional signals. Nutrient availability strongly influences defense investment: nitrogen limitation can constrain the production of nitrogen-rich metabolites, whereas excess carbon availability often promotes the accumulation of phenolic compounds such as flavonoids and lignin derivatives.^160^^,^^161^ This flexibility highlights the plasticity of plant metabolic responses, enabling optimization of defense strategies under varying resource conditions.

### 6.2. Energy-sensing pathways: TOR and SnRK1

Central to the regulation of the growth-defense trade-off are energy-sensing signaling pathways that integrate cellular metabolic status with developmental and stress responses. SNF1-RELATED KINASE 1 (SnRK1) and TARGET OF RAPAMYCIN (TOR) represent two key regulatory hubs that function antagonistically to balance growth and defense. Under conditions of nutrient and energy sufficiency, TOR promotes anabolic processes, including protein synthesis, ribosome biogenesis, and cell proliferation.^162^ Activation of TOR signaling supports growth and biomass accumulation while generally suppressing defense responses, reflecting prioritization of resource investment toward development.^95^^,^^163^^,^^164^

In contrast, SnRK1 is activated under energy-limiting or stress conditions and promotes catabolic pathways while repressing energy-intensive biosynthetic processes.^73^ Through this shift, SnRK1 enhances stress tolerance and redirects metabolic resources toward defense-associated pathways, supporting survival under adverse conditions.

The balance between TOR and SnRK1 activity is dynamically regulated and shifts toward SnRK1 activation during stress, enabling energy conservation and metabolic reallocation toward defense. This regulatory switch is closely coordinated with hormonal signaling pathways, particularly those involving jasmonic acid (JA) and abscisic acid (ABA), which further modulate energy distribution and metabolic restructuring under stress conditions.^165^^,^^166^ Together, the TOR-SnRK1 regulatory axis provides a mechanistic framework linking energy sensing to metabolic reprogramming and defense activation. By integrating environmental cues with cellular energy status, this system enables plants to balance growth and defense while maintaining metabolic homeostasis.

### 6.3. Metabolomic evidence for growth-defense trade-offs

Recent advances in metabolomics have provided strong experimental support for the metabolic basis of the growth-defense trade-off. Global metabolite profiling studies have consistently shown that activation of defense responses is associated with extensive reconfiguration of metabolic networks, including reductions in growth-associated metabolites and concomitant increases in defense-related compounds.^167-169^ Time-resolved metabolomic analyses further reveal that these changes are highly dynamic. Early stages of defense are often characterized by rapid depletion of central metabolites, followed by the accumulation of specialized metabolites during later phases. This temporal pattern reflects a sequential redistribution of resources from primary metabolism toward defense-associated pathways, highlighting the coordinated nature of metabolic reprogramming.^170^^,^^171^

Trade-offs are also evident at the level of individual metabolic pathways. For example, increased flux through the phenylpropanoid pathway can limit the availability of precursors and energy for other biosynthetic processes, illustrating competition for shared metabolic resources. Similarly, changes in amino acid metabolism during defense responses can constrain protein synthesis and growth, reinforcing the interconnectedness of metabolic networks.^172^^,^^173^

Importantly, metabolomic studies under combined or sequential stress conditions demonstrate that growth-defense trade-offs are not always linear or predictable. Instead, plants exhibit context-dependent metabolic responses, where interactions among multiple signaling pathways and environmental factors generate complex and sometimes non-additive metabolic outcomes. These findings underscore the importance of adopting systems-level approaches to understand the regulation of growth-defense interactions.

## 7. Insights from modern metabolomics technologies

Advances in metabolomics have fundamentally transformed our understanding of plant defense by enabling comprehensive, systems-level analysis of metabolic reprogramming. Unlike traditional biochemical approaches that focus on individual metabolites or pathways, modern metabolomics captures large-scale metabolite profiles, allowing simultaneous quantification of hundreds to thousands of compounds within a biological system.^5^^,^^174^ This high-throughput capability provides an integrated view of how metabolic networks respond to stress.^174^^,^^175^ Metabolomics has revealed that defense-associated metabolic reprogramming is highly dynamic and context-dependent, varying across spatial, temporal, and environmental dimensions. Time-resolved and spatial metabolomics approaches have demonstrated that early stress responses involve rapid and transient metabolite changes, followed by longer-term metabolic adjustments associated with acclimation.^10^^,^^176^

A key strength of metabolomics lies in its ability to link metabolic phenotypes with underlying regulatory mechanisms. Integration of metabolomics with transcriptomics, proteomics, and fluxomics enables reconstruction of regulatory networks connecting signaling pathways, gene expression, and metabolic outputs.^6^^,^^177^ Such multi-omics approaches facilitate identification of pathway-level regulation, metabolic bottlenecks, and candidate genes associated with defense-related traits.

Despite these advances, important challenges remain. Limitations in metabolite annotation, data standardization, and reproducibility across platforms constrain the interpretation of metabolomic datasets, particularly under complex stress conditions.^178^^,^^179^ Moreover, establishing causal relationships between metabolite changes and functional defense outcomes remains a major challenge in the field.^180^ Overall, modern metabolomics provides a powerful framework for understanding plant defense as an integrated, multi-layered process, linking molecular regulation with metabolic function and enabling the transition toward predictive models of plant stress responses.

### 7.1. Targeted and untargeted metabolomics approaches

Metabolomics strategies can broadly be classified into targeted and untargeted approaches, each providing complementary insights into plant defense-associated metabolic reprogramming. Targeted metabolomics focuses on the accurate quantification of predefined metabolites or metabolite classes, enabling high sensitivity, specificity, and quantitative reliability. This approach is particularly useful for validating known metabolic pathways and monitoring key defense-related compounds such as phytohormones, flavonoids, and phytoalexins. ^181^^,^^182^

In contrast, untargeted metabolomics aims to capture a comprehensive snapshot of the metabolome, enabling the detection of a broad range of known and unknown metabolites without prior selection. Advances in high-resolution mass spectrometry, including LC-MS and GC-MS platforms, have significantly expanded metabolome coverage, allowing detection of thousands of metabolite features in a single analysis.^174^^,^^183^^,^^184^ This approach has been instrumental in identifying stress-specific metabolic signatures and uncovering previously uncharacterized metabolic pathways involved in plant defense.

It is important to note that analytical platforms such as LC-MS and GC-MS can be applied in both targeted and untargeted modes, depending on experimental design and data acquisition strategies. LC-MS is particularly suited for semi-polar and non-volatile metabolites, whereas GC-MS is highly effective for volatile and derivatized primary metabolites, providing complementary coverage of plant metabolic networks.^185-187^ Another complementary platform is nuclear magnetic resonance (NMR)-based metabolomics, which offers high reproducibility, minimal sample preparation, and inherently quantitative measurements. However, NMR has lower sensitivity compared to MS-based approaches, limiting its ability to detect low-abundance metabolites.^188^

Despite their strengths, both targeted and untargeted approaches have limitations. Targeted analyses are restricted to known metabolites and predefined pathways, whereas untargeted approaches face challenges related to metabolite identification, data processing, and annotation accuracy. These limitations highlight the importance of integrating multiple analytical platforms and approaches to achieve comprehensive and reliable metabolomic characterization. Together, the combination of targeted, untargeted, and complementary analytical techniques provides a robust framework for dissecting plant metabolic responses to stress, enabling both hypothesis-driven and discovery-based investigations.

### 7.2. Spatial and temporal metabolomics

A key limitation of conventional metabolomics approaches is the loss of spatial and temporal resolution, which can obscure localized and dynamic metabolic responses. Recent advances in spatial and temporal metabolomics have begun to address these challenges, enabling more precise characterization of when and where metabolic changes occur within plant tissues.

Spatial metabolomics, particularly mass spectrometry imaging (MSI) techniques such as matrix-assisted laser desorption/ionization (MALDI) imaging, allows direct visualization of metabolite distributions at tissue and cellular levels. These approaches have revealed that defense-related metabolites often accumulate in highly localized patterns, reflecting site-specific activation of metabolic pathways during pathogen infection or environmental stress.^189^^,^^190^ Such spatial resolution provides critical insight into the compartmentalization of metabolic processes and their coordination with localized signaling events.

Temporal metabolomics complements these approaches by capturing the dynamic progression of metabolic responses over time. Time-course analyses have demonstrated that early stress responses are characterized by rapid changes in primary metabolism and signaling-related metabolites, followed by the accumulation of specialized defense compounds during later stages.^6^ This temporal resolution is essential for distinguishing causal metabolic events from downstream consequences and for understanding the sequence of metabolic reprogramming during stress responses. Together, spatial and temporal metabolomics provide a more comprehensive view of plant defense, revealing that metabolic reprogramming is both highly localized and dynamically regulated. These approaches are critical for linking metabolic changes to specific tissues, developmental stages, and phases of stress response, thereby advancing mechanistic understanding of plant stress adaptation.

### 7.3. Fluxomics and stable isotope labeling

While metabolomics provides information on metabolite abundance, it does not directly capture metabolic fluxes, which represent the actual rates of biochemical reactions within metabolic networks. Fluxomics, particularly when combined with stable isotope labeling, addresses this limitation by enabling quantitative analysis of pathway activity and carbon flow through metabolic systems.^170^^,^^191^ In stable isotope-based flux analysis, labeled substrates, commonly ^13C-enriched compounds, are incorporated into metabolic pathways, allowing the tracing of carbon through interconnected networks. By monitoring the incorporation of isotopic labels into downstream metabolites, it is possible to quantify pathway fluxes and determine how metabolic routes are reprogrammed under stress conditions. ^192^

This approach has provided important insights into defense-associated metabolic reprogramming. Fluxomic studies have demonstrated increased carbon allocation toward the pentose phosphate pathway, amino acid biosynthesis, and secondary metabolite production during stress, reflecting enhanced demand for reducing power, precursors, and defense compounds.^170^^,^^193^ Such analyses reveal that metabolic adjustments during defense are not limited to changes in metabolite levels but involve coordinated shifts in pathway activity.

Fluxomic analyses have also highlighted the remarkable plasticity of plant metabolic networks. Plants can rapidly redirect metabolic fluxes in response to environmental signals, enabling dynamic adjustment of energy production, biosynthesis, and defense-related pathways.^10^ This flexibility is essential for maintaining metabolic homeostasis while supporting defense responses. Integration of fluxomics with conventional metabolomics further enhances our ability to interpret metabolic reprogramming by linking metabolite abundance with pathway activity. Together, these approaches provide a more comprehensive and mechanistic understanding of plant metabolic responses to stress.

### 7.4. Multi-omics integration and computational approaches

The complexity of plant defense responses necessitates integrative approaches that combine metabolomics with other omics datasets. Multi-omics integration linking transcriptomics, proteomics, and metabolomics enables the systematic analysis of relationships among gene expression, protein abundance, and metabolite levels, providing a more comprehensive view of biological regulation.^35^^,^^179^ These datasets are commonly analyzed using correlation-based methods, network modeling, and pathway enrichment analyses to identify coordinated changes across molecular layers. Such approaches allow the reconstruction of regulatory networks that connect signaling pathways with metabolic outputs, revealing how stress-responsive genes and enzymes drive metabolic reprogramming.^194^

More recently, machine learning (ML) approaches have been increasingly applied to metabolomics and multi-omics datasets. These methods are used for tasks such as classification of stress conditions, identification of metabolic biomarkers, prediction of pathway activity, and inference of genotype-phenotype relationships.^195^^,^^196^ By capturing complex, non-linear relationships within high-dimensional datasets, ML approaches can uncover patterns that are not accessible through conventional statistical analyses.^197^ However, important limitations remain. Challenges in metabolite annotation, data standardization, and integration across platforms can affect the robustness of multi-omics analyses. In addition, machine learning models often face issues related to interpretability, overfitting, and the need for large, high-quality datasets for validation. These constraints highlight the need for careful experimental design and cross-validation when applying computational approaches to plant metabolomics.

Despite these challenges, multi-omics integration provides a powerful framework for linking molecular regulation with metabolic function. It enables the identification of key regulatory nodes and candidate pathways for metabolic engineering and crop improvement. Furthermore, integration of genotype-specific omics data allows the characterization of variation in metabolic responses, offering insights into the genetic basis of stress tolerance and supporting the development of resilient crop varieties (Table 2).

## 8. Emerging frontiers and future perspectives

Rapid advances in molecular biology, analytical technologies, and computational approaches are reshaping our understanding of plant defense and metabolic reprogramming. While significant progress has been made in characterizing metabolic responses to stress, emerging tools now offer unprecedented opportunities to resolve these processes at higher spatial, temporal, and mechanistic resolution. These developments are driving a transition from descriptive metabolomics toward predictive and engineering-oriented frameworks, with important implications for both fundamental research and crop improvement.

### 8.1. Single-cell and spatially resolved metabolomics

A major frontier in plant metabolomics is the ability to resolve metabolic processes at cellular and subcellular resolution. Conventional metabolomic approaches rely on bulk tissue analysis, which can obscure cell-type-specific metabolic heterogeneity and mask localized defense responses.^10^^,^^180^ In contrast, plant defense is often spatially restricted, with distinct cell types exhibiting specialized metabolic activities during stress.

Recent advances in single-cell and spatially resolved metabolomics are beginning to overcome these limitations. High-resolution mass spectrometry imaging (MSI) techniques, including matrix-assisted laser desorption/ionization (MALDI) imaging, along with micro-sampling approaches, enable the mapping of metabolite distributions across tissues with increasing spatial precision.^189^^,^^198^ These methods have revealed that defense-related metabolites often accumulate in highly localized patterns, reflecting cell-type-specific activation of metabolic pathways.^199^^,^^200^ Such spatial resolution provides critical insight into the organization of metabolic networks within tissues, allowing the identification of metabolic hotspots associated with pathogen invasion or stress exposure.^189^ This level of detail is particularly important for understanding how localized signaling events are translated into spatially coordinated metabolic responses.

Integration of spatial metabolomics with single-cell transcriptomics further enhances this analytical framework. By linking metabolite distributions with gene expression profiles at cellular resolution, these approaches enable direct association between regulatory programs and metabolic phenotypes.^201^ This combined strategy offers new opportunities to dissect the cellular basis of defense responses and to identify cell-specific regulatory mechanisms underlying metabolic reprogramming. Despite these advances, challenges remain, including limitations in sensitivity, metabolite coverage, and data integration across platforms.^188^ Addressing these constraints will be essential for fully exploiting single-cell metabolomics in plant defense research.

### 8.2. Synthetic biology and metabolic engineering

The expanding understanding of defense-associated metabolic pathways has opened new opportunities for engineering plant metabolism to enhance stress resilience. Synthetic biology and metabolic engineering approaches enable targeted manipulation of biosynthetic pathways to increase the production of defense-related metabolites or introduce novel metabolic functions into plants.^202^^,^^203^

Advances in pathway engineering have allowed the modification of key enzymes and regulatory nodes controlling secondary metabolism. For example, overexpression or editing of transcription factors such as MYB and bHLH families has been shown to enhance flavonoid and phenylpropanoid biosynthesis, leading to improved resistance against pathogens and environmental stress.^204^^,^^205^ Similarly, engineering terpenoid and alkaloid biosynthetic pathways has enabled the optimization of metabolite production through manipulation of precursor supply and pathway flux.^131^^,^^206^ Emerging synthetic biology tools, including modular pathway design, gene stacking, and CRISPR/Cas-based genome editing, have further expanded the capacity to reprogram plant metabolic networks. These approaches allow precise control of metabolic flux, enabling coordinated regulation of multiple pathway components and minimizing trade-offs associated with defense activation.^207^^,^^208^

In addition, integration of metabolomics with engineering strategies facilitates the identification of metabolic bottlenecks and regulatory constraints. Systems-level analyses can guide rational design of metabolic pathways by linking metabolite accumulation patterns with underlying genetic and enzymatic controls.^209^ Despite these advances, challenges remain, including pathway complexity, unintended metabolic imbalances, and potential fitness costs associated with enhanced defense metabolism.^210^^,^^211^ Addressing these limitations will require improved predictive models and tighter integration of multi-omics data to achieve efficient and sustainable metabolic engineering outcomes.

### 8.3. CRISPR-based rewiring of metabolic networks

Genome editing technologies, particularly CRISPR/Cas systems, have revolutionized plant biology by enabling precise and efficient modification of genes involved in metabolism and defense. These tools allow targeted manipulation of key regulatory nodes, including transcription factors, metabolic enzymes, and signaling components, thereby enabling direct control over defense-associated metabolic pathways.^207^^,^^212^

CRISPR-based approaches have been successfully applied to modify metabolic pathways associated with stress tolerance, including enhancement of flavonoid biosynthesis and modulation of hormone signaling pathways.^213^^,^^214^ For example, targeted editing of transcriptional regulators and biosynthetic genes has enabled fine-tuning of phenylpropanoid and secondary metabolite pathways, resulting in improved resistance to biotic and abiotic stresses.^215^^,^^216^

Beyond single-gene modifications, multiplex genome editing allows simultaneous targeting of multiple genes, enabling coordinated rewiring of complex metabolic networks.^217^^,^^218^ This capability is particularly important for plant metabolic systems, where multiple pathway components and regulatory layers must be adjusted to achieve meaningful phenotypic outcomes. In addition to engineering applications, CRISPR technologies provide powerful tools for functional genomics. Gene knockouts, knock-ins, and regulatory edits can be used to dissect gene function and uncover interactions among metabolic and signaling networks, offering deeper insight into the mechanisms underlying metabolic reprogramming.^219^

Integration of genome editing with metabolomics further enhances this framework by linking targeted genetic modifications with downstream metabolic phenotypes. This combined approach enables identification of causal relationships between gene function and metabolic outcomes, providing a robust platform for both mechanistic studies and rational metabolic engineering.

### 8.4. Artificial intelligence and predictive metabolomics

The increasing complexity and high dimensionality of metabolomics datasets necessitate advanced computational approaches for effective data analysis and interpretation. Artificial intelligence (AI) and machine learning (ML) methods have emerged as powerful tools for extracting biologically meaningful patterns from large-scale metabolomics and multi-omics datasets.^179^^,^^195^

Machine learning algorithms have been applied to a range of analytical tasks in plant metabolomics, including classification of stress conditions, identification of metabolic biomarkers, prediction of pathway activity, and modeling of genotype-phenotype relationships.^220^^,^^221^ For example, supervised learning approaches such as random forests and support vector machines are widely used for stress classification and biomarker discovery, while unsupervised methods such as clustering and dimensionality reduction help reveal underlying metabolic structures in complex datasets.

Integration of metabolomics with transcriptomics and proteomics using ML-based frameworks enables the reconstruction of predictive models that link gene regulation to metabolic outputs. These approaches facilitate identification of key regulatory nodes and metabolic pathways associated with stress tolerance, thereby supporting hypothesis generation and guiding experimental validation.

However, several limitations constrain the application of AI in plant metabolomics. Challenges include incomplete metabolite annotation, variability across datasets, and limited availability of standardized, high-quality training data. In addition, many ML models suffer from limited interpretability, making it difficult to establish causal relationships between predicted features and biological function.^222^^,^^223^ Despite these challenges, AI-driven metabolomics holds significant potential for advancing predictive biology in plants. When combined with robust experimental validation, these approaches can support the development of predictive models for stress responses and contribute to rational strategies in metabolic engineering and crop improvement.

### 8.5. Translational perspectives for crop improvement

Translating insights from metabolic reprogramming into practical applications represents a major objective of plant science research. Understanding how metabolic networks are regulated under stress provides a foundation for developing crop varieties with enhanced resilience to environmental challenges. One promising approach is metabolite-assisted breeding, in which metabolomic data are used to identify biochemical markers associated with desirable traits. Metabolic markers linked to stress tolerance can be integrated into breeding programs to improve selection efficiency and accelerate the development of resilient genotypes.^224^^,^^225^

Compared with traditional phenotypic selection, this strategy enables more precise identification of functional traits associated with stress adaptation. Integration of metabolomics with genomics, transcriptomics, and phenotypic data further enhances breeding strategies by enabling systems-level analysis of genotype–phenotype relationships. Such multi-omics frameworks facilitate the identification of key regulatory genes and metabolic pathways underlying stress tolerance, providing targets for both conventional breeding and biotechnological interventions.^168^^,^^180^ In addition, advances in precision breeding and genome editing technologies offer new opportunities to directly modify metabolic pathways associated with stress responses. Targeted manipulation of metabolic networks, informed by multi-omics analyzes, allows optimization of resource allocation, defense metabolism, and stress resilience in crops.^226-228^

Despite these advances, translating metabolic insights into field performance remains challenging due to environmental variability, metabolic complexity, and potential trade-offs between growth and defense. Addressing these challenges will require integration of metabolomics with field-based phenotyping and environmental data. Overall, the combination of metabolomics, multi-omics integration, and precision breeding strategies provides a powerful framework for developing climate-resilient crops and advancing sustainable agriculture.

## 9. Conclusions

Metabolic reprogramming represents a central and unifying feature of plant defense, enabling the coordinated integration of signaling networks, gene regulation, and biochemical pathways in response to environmental stress. Rather than operating as isolated processes, primary and secondary metabolism function as interconnected and dynamically regulated networks, controlled by phytohormones, transcriptional regulators, and energy-sensing pathways such as TOR and SnRK1. This systems-level perspective highlights metabolism not only as a source of energy and biosynthetic precursors but also as an active determinant of defense outcomes.

Advances in metabolomics have been instrumental in uncovering the complexity, plasticity, and context dependence of these responses. By enabling large-scale, high-resolution profiling of metabolites, metabolomics has revealed how metabolic networks are reconfigured across different stress conditions, tissues, and developmental stages. Integration with transcriptomics, proteomics, and fluxomics has further facilitated the reconstruction of regulatory networks linking molecular mechanisms with metabolic phenotypes. Together, these approaches are shifting the field from largely descriptive frameworks toward mechanistic and increasingly predictive models of plant defense.

Despite these advances, important challenges remain. A key priority is to resolve the spatial and temporal dynamics of metabolic reprogramming, particularly under combined or sequential stress conditions that more accurately reflect natural environments. In addition, distinguishing causal defense-related metabolites from general stress-associated changes and improving metabolite annotation remain significant limitations. Addressing these challenges will require continued development of high-resolution analytical technologies, flux-based approaches, and robust computational frameworks.

From a translational perspective, understanding how metabolic networks are coordinated with growth and resource allocation provides a foundation for developing crops with enhanced stress resilience. Integration of metabolomics with genome editing, synthetic biology, and precision breeding offers promising strategies to optimize metabolic pathways and improve plant performance. However, successful application will depend on balancing defense enhancement with growth and yield, highlighting the importance of the growth-defense trade-off.

In conclusion, a mechanistic and systems-level understanding of metabolic reprogramming integrating signaling, gene regulation, and metabolomics provides a powerful framework for advancing plant biology. Such knowledge is essential for addressing global challenges in agriculture by enabling the development of resilient crops capable of maintaining productivity under increasingly variable environmental conditions.

## Data Availability

No new data was generated for this study.
